# Analyzing the Attitudes of Teachers in Spain Toward Stuttering

**DOI:** 10.1111/1460-6984.70223

**Published:** 2026-03-26

**Authors:** Marta Modrego‐Alarcón, María Cruz Pérez‐Yus, Mayte Navarro‐Gil, Héctor Morillo‐Sarto, Alicia Monreal‐Bartolomé, Kenneth O. St. Louis

**Affiliations:** ^1^ Departamento de Psicología y Sociología Universidad de Zaragoza Zaragoza España; ^2^ West Virginia University Morgantown West Virginia USA

**Keywords:** attitudes, public opinion survey of human attributes–stuttering (posha–s), Spain, stuttering, teachers

## Abstract

**Introduction:**

Teachers play a crucial role in fostering supportive environments for all students. However, research indicates that their knowledge and attitudes toward stuttering are often similar to those of the general population. The primary aim of this study is to examine the attitudes of teachers at different educational levels in Spain toward stuttering and to explore how these attitudes relate to specific sociodemographic variables.

**Method:**

A cross‐sectional, self‐report design was employed with a sample of 250 teachers from various educational levels and teaching roles across Spain. Participants completed the Public Opinion Survey on Human Attributes‐Stuttering (POSHA–S).

**Results:**

Overall, the attitudes of teachers in Spain toward stuttering were generally positive and exceeded those observed in other comparison groups, except for the *Knowledge Source* component. Positive attitudes toward stuttering were strongly associated with factors reflecting experience and knowledge, such as training in stuttering, teaching students who stutter, current teaching role, and self‐identification as a person who stutters.

**Conclusion:**

Our findings highlight the importance of providing teachers with targeted training and direct experience with students who stutter to foster supportive attitudes.

**WHAT THIS PAPER ADDS:**

*What is already known on the subject*

- Attitudes toward stuttering can significantly affect the wellbeing and development of people who stutter. While these attitudes have been studied internationally, no research has examined teachers’ attitudes in Spain toward students who stutter.

*What this paper adds to existing knowledge*

- This study shows that teachers in Spain—including School‐Based Speech‐Language Pathologists (SLPs) and Special Education Teachers—generally hold more positive attitudes than those reported in previous studies. However, some harmful beliefs remain, such as underestimating genetic factors or perceiving students who stutter as shy, introverted or incapable of demanding tasks.

*What are the potential or actual clinical implications of this work?*

- The lack of experience, training and information about stuttering, along with the better attitudes of specialized teachers, highlights the need to prioritize stuttering in the SEN agenda to address biased beliefs and reactions toward students who stutter.

## 1 Introduction

Stuttering affects not only the fluency of speech, but also the quality of life of people who stutter, especially in the vitality, social functioning, emotional functioning and mental health status domains (Craig et al. 2009). Compared to nonstuttering peers, children and adolescents who stutter experience increased anxiety and depressive symptoms (Bernard et al. 2022; Briley et al. 2021), as well as increased rejection and bullying by classmates (Berchiatti et al. 2020; Langevin et al. 2009; Plexico et al. 2013). Relationships with teachers and peers, as well as academic performance at school, may be affected by stuttering (Klompas and Ross 2004). University students who stutter also report stories of dissatisfaction and lost opportunities in terms of social interactions, classroom engagement and performance (Meredith and Packman 2015).

The public view of stuttering is generally unfavourable. This fact has been widely documented across multiple age groups (Al‐Khaledi et al. 2009; Ezrati‐Vinacour et al. 2001; Flynn and St. Louis 2011), different professions (e.g., Abdalla and St. Louis 2012; Maviş et al. 2013) or diverse geographic locations (e.g., Arafa et al. 2021; Ip et al. 2012; St. Louis and Roberts 2013; St. Louis et al. 2016).

Widespread awareness of public attitudes toward people who stutter is imperative to develop strategies to reduce the impact that stereotypes have on their quality of life and overall health (Lefort et al. 2021). Teachers have the potential to mitigate negative experiences and create more comfortable and understanding environments (Arnold et al. 2015; Hearne et al. 2021; Plexico et al. 2013; Schwab and Rossmann 2020; Werle and Byrd 2021). Although teachers are often assumed to have satisfactory knowledge and encouraging attitudes toward their students with special needs, including those who stutter, previous literature indicates that teachers hold unsubstantiated beliefs about the causes of stuttering and pervasive negative stuttering stereotypes that could place students who stutter at an obvious disadvantage (Crowe and Walton 1981; Woods and Williams 1976; Yeakle and Cooper 1986).

Much of the latest literature that assesses teachers' public attitudes toward stuttering has used a standardized instrument known as the *Public Opinion Survey on Human Attributes‐Stuttering* (*POSHA–S*) 2011). The *POSHA–S* has been shown to have satisfactory internal consistency and is reliable, valid, and translatable, facilitating the comparison of its results across different studies (e.g., St. Louis 2012, 2014). Abdalla and St. Louis (2012) found that 471 Arab public‐school teachers, despite recognizing common stuttering traits and knowing people who stutter, were largely misinformed about its causes, held personality stereotypes and reported unsupported coping strategies. Abrahams et al. (2016) found that 469 African primary school teachers had knowledge gaps and held personality stereotypes about stuttering, but showed more positive attitudes regarding the potential of people who stutter and their reactions to it. Other studies using *POSHA–S* have not found large or significant differences in attitudes toward stuttering between the teaching and non‐teaching population (Arnold et al. 2015; Junuzović‐Žunić et al. 2023; Li and Arnold 2015). A recent study that analyzed nearly all the potential predictors of the *POSHA–S* from a database of more than 22 000 respondents found that summary scores for samples of teachers were only very slightly more positive than for the general public (St. Louis 2024a).

The literature referring to university professors is more limited and uses other assessment instruments. A study conducted by Dorsey and Guenther (2000) found that professors and university students perceive students who stutter as being more negative on most personality traits, compared to how they perceive non‐stuttering university students. Surprisingly, the professors turn out to be even more negative in their attitudes. The university student´s own experience appears to be consistent with this idea (Werle and Byrd 2021).

The landscape offered by the factors that contribute to teachers’ attitudes towards stuttering is heterogeneous, with some factors unstudied. These heterogeneous results can be the result of the diverse sample compositions with regard to regional and cultural variations as well as the specific teaching sample characteristics. Our goal is to enrich the field's state by clarifying the role of these relevant factors. That is the case for the role of sex favouring better attitudes in the case of women, with studies showing partial or no relationship (Arnold et al. 2015; Fichman et al. 2025; Li and Arnold 2015; Veerabhadrappa et al. 2025). This uncertainty holds also true for age and years of teaching experience, with studies showing positive (Almudhi 2022; Arnold et al. 2015; Li and Arnold 2015) or no relationship (Fichman et al. 2025; Valente et al. 2017; Veerabhadrappa et al. 2025). However, years of education do seem to exert a positive effect on teachers’ attitudes towards stuttering (Almudhi 2022; Arnold et al. 2015; Fichman et al. 2025; Li and Arnold 2015; Veerabhadrappa et al. 2025).

Experience with students who stutter usually exerts a positive influence (Abdalla and St. Louis 2012; Irani et al. 2012; Yeakle and Cooper 1986), although a recent study has not found this relationship (Veerabhadrappa et al. 2025). Consensus is more unambiguous in the case of the positive impact of training on stuttering (Arnold and Li 2016; Crowe and Walton 1981; Hearne et al. 2021; Panico et al. 2017; Yeakle and Cooper 1986). However, the scarcity of studies that have included specialist teachers (e.g., School Counsellors, School‐Based Speech‐Language Pathologists (SLPs) and Special Education Teachers), in addition to other traditionally included teaching roles (e.g., Early Childhood Education, Primary and High School Education) have not shown better attitudes towards stuttering (Lee 2014; Maviş et al. 2013).

The results of studies of teacher attitudes toward stuttering underscores the need to offer stuttering training and resources for teachers in order to dispel misconceptions about stuttering and, thereby, to enable greater support for students who stutter, both in schools (Abdalla and St. Louis 2012; Hearne et al. 2021; Jenkins 2010) and universities (Azios et al. 2022; Daniels et al. 2011; Werle and Byrd 2021).

St. Louis (2024b) found that different countries of the world were the strongest predictor of differences in stuttering attitudes, which were closely related to different languages and regions (e.g., North America or South Asia). To date, there are no published *POSHA–S* studies of teacher attitudes from Spain, indicating an important gap in the literature. Even though Western European summary attitudes, on average, were found to be among the most positive attitudes observed so far on the *POSHA–S* (St. Louis 2024b), wide differences have been found within various countries (St. Louis et al. 2016).

To address the need to sample teacher attitudes at various education levels, the main objective of this study was to examine the attitudes of teachers across different educational levels in Spain toward stuttering and to compare these attitudes with public attitudes documented in the *POSHA–S* database. Additionally, we aimed to explore whether various sociodemographic factors, including demographic characteristics and education and experience related to stuttering, are associated with the attitudes of teachers in Spain toward this condition, as well as the explanatory power of these variables.

## 2 Methods

### 2.1 Design

A cross‐sectional self‐report design was used, with a sample of teachers from various educational levels throughout Spain. Purposive sampling was used to identify potential study participants.

### 2.2 Participants

Data were collected across all regions of Spain. Teachers were eligible to participate in the research if they met the following inclusion criteria: (a) be teachers of any official educational centre regardless of teaching role or educational level, including early childhood, primary/elementary, high school, vocational training, official language school, conservatory or arts education, adult education centres, universities or school counsellors; (b) be actively working; (c) be between 18 and 70 years of age; (d) be fluent in Spanish and (e) consent voluntarily to participate in the study.

It was calculated that a minimum sample of 235 teachers (with a significance level of 0.05 and a statistical power of 90%) would be needed to analyze attitudes toward stuttering and knowledge and self‐reactions toward stuttering, respectively. The minimum calculated sample size was reached, obtaining a total of 250 participants.

### 2.3 Procedure

Potential participants were reached through multiple channels. An email was sent to numerous and diverse educational centres throughout Spain in which teachers were asked to participate. In addition, it was advertised on social networks, and respondents were encouraged to forward it to other participants following a snowball sample collection method. Data was registered between May and June 2023.

### 2.4 Instruments

Two instruments were utilized in the study:

#### 2.4.1 Sociodemographic Variables Questionnaire

The instrument gathered information on participants’ demographics (age, sex, birthplace, years of completed studies [total number of formal academic years completed], marital status) and professional background (teaching experience, type of school and current teaching role). Finally, participants were asked about their teaching experience with students who stutter, any training they had received on stuttering, and whether they believed such training should be provided, using a 5‐point Likert scale.

#### 2.4.2 The Public Opinion Survey of Human Attributes‐Stuttering (POSHA–S)

As noted, the *POSHA–S* is a standardized instrument that measures public attitudes toward stuttering (St. Louis 2011). This study used a Spanish version of the *POSHA–S*. Following a process similar to other studies (e.g., Przepiorka et al. 2013) the first and corresponding authors, both native Spanish speakers, independently translated the survey and refined it collaboratively. A native translator back‐translated it into English to compare with the original, revealing no significant differences in meaning. The final Spanish version was tested with ten teachers, who reported no issues with item comprehension or the survey procedure. The *POSHA–S* is composed of three sections: (1) a demographic section; (2) a general section comparing stuttering with four other attributes (intelligent, left‐handed, mentally ill and obese) and (3) a detailed section on stuttering. All scale ratings are converted to a range of –100 to +100 scale. Lower scores reflect less accurate, sensitive or worse informed attitudes and higher scores reflect more accurate, sensitive or well‐informed attitudes. The *POSHA–S* is evaluated by calculating means for each item, means of groups of items that represent different components, which are then averaged into three subscores. Two subscores focus on stuttering, and one is dedicated to obesity and mental illness. The *Overall Stuttering Score (OSS)* is obtained by averaging the two subscores related to stuttering, *Beliefs* and *Self‐Reactions* (St. Louis 2011). A *POSHA–S* database of samples around the world permits comparison of individual samples to what can be regarded as a worldwide average (St. Louis 2024a). Typically, an investigator's sample means are compared to the database average (i.e., median of all the sample means to reduce the effect of outlier samples) and the percentile ranking of the investigator's means relative to all the database sample means. At the time of this study, 230 samples representing 20 941 respondents from 48 countries and 30 languages comprised the comparison average.

### 2.5 Data Analysis

Data was analyzed with the Statistical Package for Social Sciences (SPSS 25.0).

Descriptive statistics were calculated for sociodemographic variables, including means and standard deviations for continuous variables and frequencies and percentages for categorical variables. To examine differences in POSHA‐S scores, univariate analyses were conducted. For comparisons between two groups, Student's *t*‐tests were used when data met normality assumptions, and Mann‐Whitney U tests when they did not. For comparisons among three or more groups, one‐way ANOVAs were used, with Kruskal–Wallis tests as the non‐parametric alternative when assumptions of normality or homogeneity of variance were violated. Pearson correlations were used to assess linear associations between continuous variables, and Spearman correlations were applied when variables did not meet parametric assumptions.

Three multiple linear regression models were tested, including as explanatory variables the sociodemographic variables and as criterion variable (dependent variable) the OSS, Beliefs and Self‐Reactions, respectively, in each model. The sociodemographic variables included: self‐identification as a person who stutters, having received training in stuttering, having teaching experience with students who stutter, teaching experience (years), current teaching role, age, sex, years of completed studies and familiarity with people who stutter (categorized according to Arnold et al. 2015). Current teaching role was grouped for analysis based on professional training and likely exposure to communication disorders, which were considered most relevant to attitudes toward stuttering (i.e., (1) Other types of teaching, (2) School Counselors, and (3) School‐Based Speech‐Language Pathologists [SLPs] and Special Education Teachers).

Significant explanatory variables were entered into stepwise multiple regression models to identify the best combination of predictors for *OSS, Beliefs* and *Self‐Reactions* scores. Model fit was assessed using Adjusted R‐squared (Adjusted *R* ^2^). An ANOVA of the regression model was conducted to test if the estimation of the dependent variable was significantly improved. For each explanatory variable, the coefficients of the regression model and the t scores were calculated to ensure that the variable contributed significantly to the regression model. The variance‐inflation factor (VIF) and the tolerance were calculated in each model to assess the assumption of non‐multicollinearity.

### 2.6 Data Sharing

The data that support the findings of this study are available from the corresponding author upon reasonable request.

## 3 Results

### 3.1 Sociodemographic Information

Table 1 presents the sociodemographic results for the 250 teachers who participated in the study (column 2). The average age of the sample was 46.33 years (SD = 11.04), with a greater proportion of women (*n* = 159, 63.6%). The mean number of years of teaching experience was 16.55 (SD = 10.94). There was a variety in the current teaching role (self‐reported). Most participants lacked teaching experience with people who stutter (67.6%) and had not received related training (74.8%), yet the majority agreed (61.6%) or strongly agreed (24.8%) that such training should be provided.

**TABLE 1:** Sociodemographic characteristics and POSHA–S scores for teachers in Spain compared with the POSHA–S database (St. Louis 2024a).

Sociodemographic variables	Spanish sample data	*POSHA–S* database average	Spanish sample percentile^a^
Sample size	250	60	92
Completion time: mean (min)	10.12	11	38
Age: mean (yr) (SD)	46.3 (11.04)	36.9	92
Born in Spain (% of total)	95.6	‐^b^	‐^b^
Years of completed studies: mean (yr) (SD)	17.8 (2.26)	14.3	92
Sex: females (% of total)	63.6	63.3	62.2
Married (% of total)	71.6	53	73
Parent (% of total)	60.8	49	68
Teaching experience: mean (yr) (SD)	16.55 (10,94)	‐^b^	‐^b^

Type of educational centre (%)			
Private	5.6	‐^b^	‐^b^
Charter	8	‐^b^	‐^b^
Public	86.4	‐^b^	‐^b^

Current teaching role (%)			
Early childhood education teacher			
Primary school teacher	9.2	‐^b^	‐^b^
High school teacher	13.2	‐^b^	‐^b^
Teacher of vocational training cycles	18.4	‐^b^	‐^b^
OLS teacher	12	‐^b^	‐^b^
Conservatory or artistic education teacher	6.8	‐^b^	‐^b^
Teacher at an adult education centre	2.8	‐^b^	‐^b^
University teacher	3.6	‐^b^	‐^b^
School counsellor	21.6	‐^b^	‐^b^
School‐based speech‐language	5.6	‐^b^	‐^b^
Pathologists (SLPs) and special education teachers	6.8	‐^b^	‐^b^
Teaching experience with students who stutter: *n* (%)	67,6	‐^b^	‐^b^
Received training in stuttering: *n* (%)	78.4	‐^b^	‐^b^
Have received training in stuttering: totally agree (%)	61.6	‐^b^	‐^b^
Income score (−100 to +100)	22	0	89
Income: Family/Friend	21	8	89
Income: Countrymen	22	0	88
Know >1 language (% responding)	77.6	53	71

Self‐identification (% total)			
Stuttering	3.6	0.3	83
Mentally ill	3.2	1	70
Obese	12.8	7	79
Left‐handed	6.4	8	38
Intelligent	28	29	46
Self‐rating health and abilities (Mean: −100 to +100)	51	54	39
Physical health	39	42	32
Mental health	45	55	23
Ability to learn	61	57	59
Speaking ability	58	62	37
Self‐rating of life priorities (Mean: −100 to +100)	47	49	43
Be safe/secure	84	82	57
Be free	82	64	96
Spend time alone	71	39	99
Attend social events	19	11	63
Imagine new things	59	33	89
Help less fortunate	61	53	76
Have exciting experiences	−43	−16	13
Practice my religion	−47	26	8
Earn money	43	60	17
Do job/duty	84	76	79
Get things done	73	76	48
Solve big problems	78	72	77

Abbreviation: OLS, Official Language School.

^a^ Percentiles held by teachers in Spain calculated from the *POSHA–S* database (St. Louis 2024a).

^b^ It is not possible to calculate the median of the specific variables in our study relative to the *POSHA–S* database.

Table 1 also compares these results to data from the 230 different samples that comprise the *POSHA–S* database (St. Louis 2024a). Column 3 presents the median, or middle sample mean, from the database, while column 4 shows percentiles for the Spanish sample generated from the *POSHA–S* database.

### 3.2 Teachers in Spain´s Attitudes Toward Stuttering

Table 2 presents the *POSHA–S* scores, including the items, components, subscores and *Overall Stuttering Score (OSS)* for teachers in Spain (column 2). It also shows the average values (median of all sample means) in the *POSHA–S* database (St. Louis 2011, 2024a) (column 3) and the percentiles for the teachers in Spain relative to all of the *POSHA–S* database samples (column 4). The teachers’ *OSS* of 32 (81st %ile) is well above the database average of 18. This score is calculated by averaging the *Beliefs subscore* (M = 53, 85th %ile) and *Self‐Reactions* subscore (M = 11, 66th %ile).

**TABLE 2:** Mean ratings for the Overall Stuttering Score (OSS), subscores, components and items by teachers in Spain, average values from the *POSHA–S* database samples, and percentiles for teachers in Spain relative to the *POSHA–S*.

*POSHA–S* summary scores	Spanish sample mean rating	*POSHA–S* database average rating	Spanish sample percentile^a^
Number	250	60	92
Overall stuttering score (OSS)	32	18	81
Beliefs	53	33	85
Traits/Personality	42	21	84
Prone to self‐blame^b^	90	84	63
Nervous or excitable^b^	31	−3	78
Shy or fearful^b^	6	−17	76
Who should help stuttering	29	16	66
Speech‐language pathologist	97	95	69
Others who stutter	21	−4	67
Medical doctor^b^	−31	−39	51
What causes stuttering	61	33	90
Genetic	−6	18	13
Learning or habit^b^	50	26	88
Emotionally traumatic experience^b^	61	−2	95
Act of god^b^	98	62	92
Virus/Disease^b^	68	39	81
Ghost/Demon/Spirit^b^	94	90	65
Potential social professional success	80	67	78
Can make friends	100	93	74
Lead a normal life	96	89	67
Do any job they want	80	54	85
Should have a good judgement job	44	42	47
Self‐reactions	11	3	66
Accommodating/Helping	64	44	83
Try to act normally	89	84	66
I should help	2	−29	72
Fill words^b^	66	35	77
Say ‘slow down’ or ‘relax’^b^	52	13	71
Make a joke^b^	90	90	45
Should hide stuttering^b^	87	76	70
Social distance/Sympathy	41	19	92
Feel comfortable or relaxed	38	37	51
Feel pity for the person who stutters^b^	59	16	91
Feel impatient^b^	66	63	50
Concern if doctor stuttered^b^	86	46	94
Concern if neighbour stuttered^b^	96	77	81
Concern if sibling stuttered^b^	55	7	78
Concern if I stuttered^b^	0	−32	76
Overall impression person who stutters	23	2	82
Want to be a person who stutters	−60	−65	71
Knowledge/Experience	−27	−34	59
Amount known	−23	−31	53
Persons known who stutter	−81	−86	72
Source: personal experience	22	18	60
Knowledge source	−33	−11	9
TV/Radio	−29	12	14
Print	−28	−14	29
Internet	−49	−27	16
School	−4	−8	42
Specialists	−55	−31	17
Obesity/Mental illness	−23	−35	83
Impression	4	−15	84
Obesity	3	−19	85
Mental illness	5	−15	76
Want/Have	−79	−79	61
Obesity	−78	−78	63
Mental illness	−81	−80	55
Amount known	5	−9	72
Obesity	9	−1	62
Mental illness	2	−21	77

^a^ Percentiles held by teachers in Spain calculated from the *POSHA–S* database (St Louis 2024a).

^b^ The signs of the mean scores for this item have been reversed to follow the same direction as the other items, so that higher scores reflect ‘better’ attitudes and lower scores reflect ‘worse’ attitudes.

The *Beliefs* subscore stands out for demonstrating higher scores in both the overall score and its four components, which reflects more accurate beliefs of participants. Most individual items within this subscore are positive, although a few exceptions are noteworthy. In the *What causes stuttering* component, participants are unlikely to attribute stuttering to genetic causes (M = –6, 13rd %ile).

Regarding the *Self‐Reactions* subscore, the Spanish sample's scores were higher in three of the four components: *Accommodating/Helping*, *Social Distance/Sympathy* and *Knowledge/Experience* (M = 64, 83th %ile; M = 41, 92nd %ile and M = –27, 59th %ile, respectively), not so for the *Knowledge Source* component (M = –33, 9th %ile); specifically, the sources of knowledge of the participants in this sample were below average in relation to school, publications, specialists, Internet and TV/radio. The *Accommodating/Helping* component reflects more sensitive attitudes toward people who stutter and stuttering compared to average attitudes in the database. Results indicate that, in an interaction with people who stutter, participants would be less likely to complete their words (M = 66, 77th %ile), to say phrases such as ‘slow down’ or ‘relax’ (M = 52, 71st %ile) or to consider that they should hide their stuttering (M = 87, 70th %ile). Furthermore, although the mean score is close to neutrality (M = 2), participants show a greater willingness to help people who stutter (72nd %ile). Similarly, the *Social Distance/Sympathy* component reflects more positive affective reactions toward people who stutter and stuttering. Participants in the Spanish sample are less likely to feel sorry or pity for the person who stutters (M = 59, 91th %ile), as well as to show concern if the person who stutters is a doctor, neighbour, sibling or oneself (M = 86, 94th %ile; M = 96, 81st %ile; M = 55, 78th %ile; M = 0, 76th %ile; respectively). Also, the overall impression of people who stutter is more positive (M = 23, 82n^d^ %ile). Finally, with regard to the *Knowledge/Experience* component, the item relating to known people who stutter stands out, given that, despite having a low absolute value (M = –81), it has a relatively high value compared to the database, standing at the 72nd %ile. The item relating to the amount of knowledge about stuttering shows a mean score of M = –23, standing at the 53rd %ile.

**FIGURE 1:**
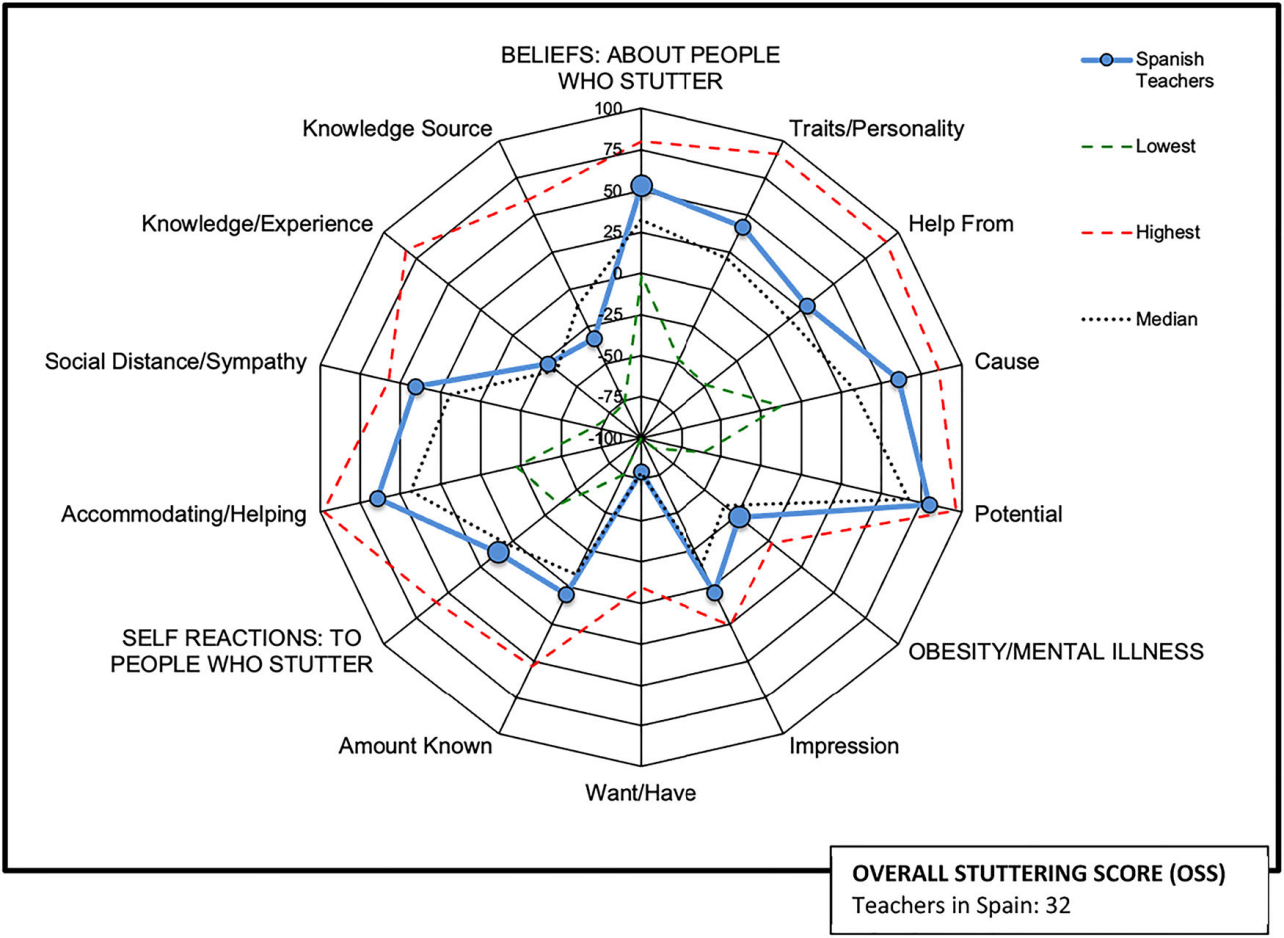
Summary of POSHA‐S radial graph for the teachers in Spain sample in comparison to the lowest, average and highest scores in the POSHA‐S database (St. Louis 2024a).

Figure 1 shows the teachers in Spain data through a radial graph, which provides a visual display of the components and subscores along with a numerical *OSS*. These data are compared with the maximum, minimum and average (median of all sample means) scores observed to date for the sample that constitutes the *POSHA–S* database (St. Louis 2011, 2024a).

### 3.3 Comparisons of Teachers' Attitudes in Spain Toward Stuttering According to Sociodemographic Variables

**FIGURE 2:**
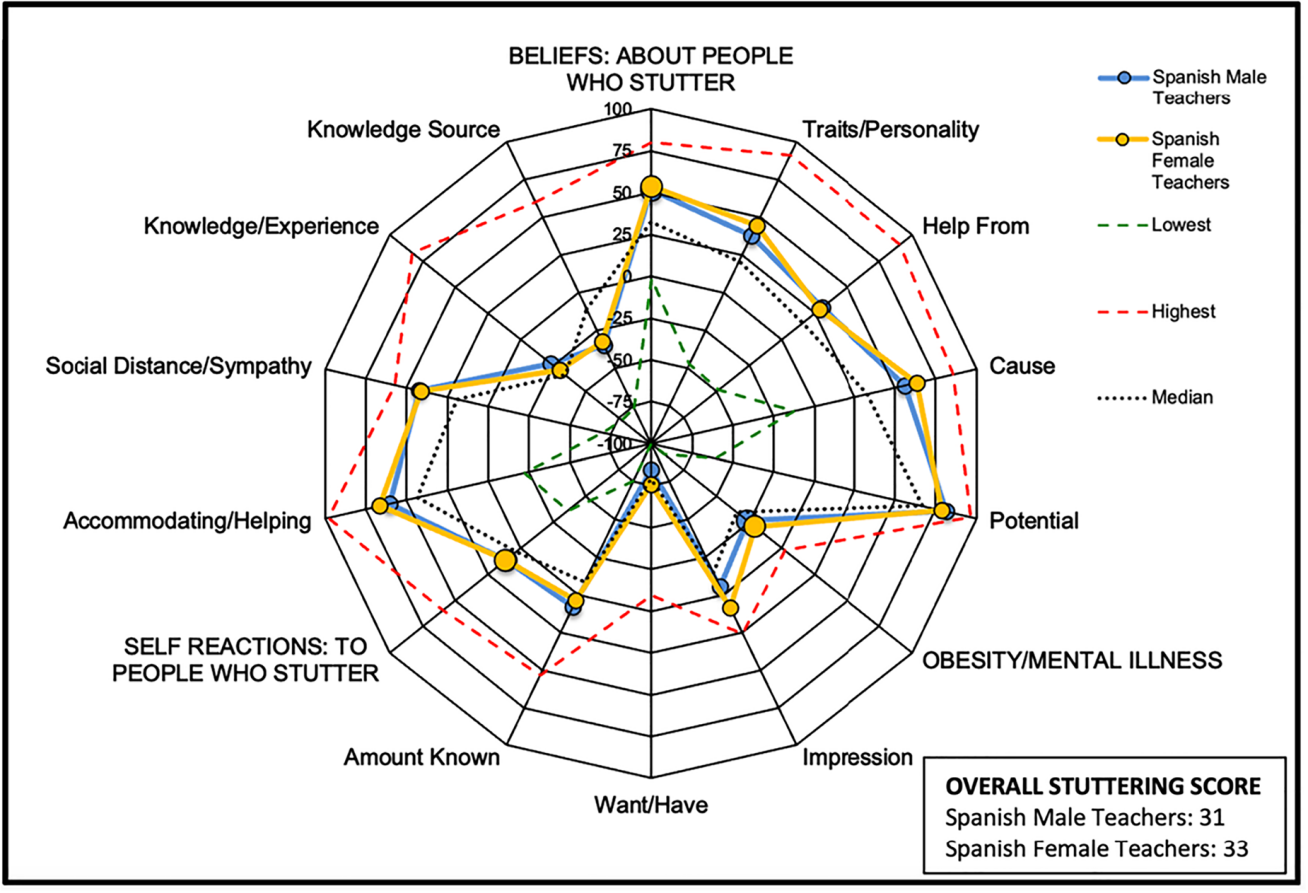
Comparison of POSHA–S scores by gender among teachers in Spain, in contrast with the lowest, average and highest scores from the POSHA–S database (St. Louis 2024a).

No statistically significant sex differences were found for the *OSS* (*p* = 0.545) or the *Self‐Reactions* subscore (*p* = 0.962), with men and women showing similar mean values (Figure 2). Non‐parametric analyses also indicated no differences in the *Beliefs* subscore (*p* = 0.181). However, women presented significantly more accurate or positive attitudes in the *Cause* (*p* = 0.009) and *Accommodating/Helping* (*p* = 0.040) subscores (Table S1, Supporting Information 1).

Regarding age, education and teaching experience, correlation analyses (Pearson and Spearman) revealed few significant associations. Age correlated negatively with the *Help from* component (*r* = −0.185; *p* = 0.003) and positively with *Knowledge/experience* (*r* = 0.156; *p* = 0.014). Years of education were negatively correlated with the *Cause* component (*r* = −0.156; *p* = 0.014), indicating that higher education levels were associated with lower accuracy in causal attributions. Teaching experience correlated positively with *Knowledge/experience* (*r* = 0.175; *p* = 0.006), suggesting greater accuracy in self‐perceived knowledge of stuttering with more years of teaching experience. All observed correlations were of low magnitude (Table S2).

Comparisons by teaching experience with students who stutter and training in stuttering (Student's *t* and Mann–Whitney U; Table S3) showed that teachers without experience or training obtained significantly lower *OSS, Self‐Reactions* and *Knowledge/experience* scores, among others (all *p* < 0.001). Participants identifying themselves as stutterers reported higher *OSS* (*p* = 0.017) and *Beliefs* (*p* = 0.003) scores.

Finally, an ANOVA by current teaching role (Other types of teaching, School Counsellors and School‐Based Speech‐Language Pathologists [SLPs] and Special Education Teachers) revealed significant group differences for *OSS* (F = 7.818; *p* = 0.001) and *Self‐Reactions* (F = 9.623; *p* < 0.001), with counsellors and specialists showing more positive attitudes. Kruskal–Wallis tests also identified significant effects for *Cause* (*p* = 0.021), *Accommodating/Helping* (*p* = 0.005) and *Knowledge Source* (*p* < 0.001), with higher scores among specialists and counsellors compared with other teachers (Table S4).

### 3.4 Explanatory Power of the Sociodemographic Variables on OSS, Beliefs and Self‐Reactions

Regarding the multiple regression analysis, the variable that best explained the *OSS* was ‘Received training in stuttering’, followed by ‘Teaching experience with students who stutter’ and ‘Self‐identifying as a person who stutters’. The remaining variables were not included in the final model because they did not significantly increase their explanatory power. For the multiple regression model tested, with three independent variables, 14% of the variance of the *OSS* was explained. The ANOVA of the regression model showed a significant effect (F (3, 249) = 12.990; *p* < 0.001). Regarding the coefficients of the regression model, *t*‐tests indicated that the variables included by the method contributed significantly to the regression model. The VIF, as well as the tolerance value, indicated that the assumption of non‐multicollinearity was met (Table 3).

**TABLE 3:** Multiple linear regressions for OSS, and Beliefs and Self‐Reactions subscores.

Significant variables	*R* ^2^	B	*β*	*t*	Tolerance	VIF	F
OSS							
Received training	0.126	9.415	0.261	4.266^***^	0.940	1.064	12.990^***^
Teaching experience with students who stutter		5.121	0.161	2.643^**^	0.943	1.061	
Self‐identifying as people who stutter		10.885	0.136	2.300^*^	0.997	1.003	

Beliefs							
Self‐identifying as a person who stutters	0.025	14.879	0.170	2.714^**^	1.000	1.000	7.366^**^

Self‐Reactions							
Received training	0.148	11.218	0.207	3.105^**^	0.768	1.302	15.409^***^
Teaching experience with students who stutter		9.732	0.205	3.395^***^	0.943	1.061	
Current teaching role		5.861	0.142	2.175^*^	0.803	1.245	

Abbreviations: *β*, standardized coefficient; B, non‐standardized coefficient; *R* ^2^, Adjusted *R* ^2^; VIF, variance inflated factor.

^*^ *p* < 0.050; ^**^ *p* < 0.010; ^***^ *p* < 0.001.

The *Beliefs* subscore was explained only by ‘Self‐identifying as a person who stutters’. For the multiple regression model tested, with one independent variable, 3% of the variance of *Beliefs* was explained. The ANOVA of the regression model showed a significant effect (F (1, 249) = 7.366; *p* = 0.007). The variable that better explained the *Self‐Reactions* subscore was ‘Received training in stuttering’, followed by ‘Teaching experience with students who stutter’ and ‘Current teaching role’. For the multiple regression model with three independent variables, 16% of the variance of *Self Reactions* was explained. The ANOVA of the multiple regression model showed a significant adjustment (F = 33.18; *p* < 0.001). For the coefficients of the multiple regression model, the *t* scores indicated that the variables considered contributed significantly to explaining the variance of *Self Reactions*. The VIF as well as the tolerance values indicated that the assumption of non‐multicollinearity was met (see Table 3).

## 4 Discussion

### 4.1 Teachers in Spain´s Attitudes Toward Stuttering

In this study, we analyzed the attitudes toward stuttering of 250 teachers from various educational levels in Spain using *POSHA–S*. Overall, our sample of teachers held better attitudes toward stuttering than previous studies (Abdalla and St. Louis 2012; Abrahams et al. 2016; Junuzović‐Žunić et al. 2023; Lefort et al. 2021) for all three subscores of the *POSHA–S*, that is, *Beliefs*, *Self‐Reactions* and *Obesity/Mental Illness*, placing them in the fourth or highest quartile of the *POSHA–S* database. This finding seems to contradict previous literature showing that teachers' perceptions toward stuttering did not differ from the non‐teaching population (Abdalla and St. Louis 2012; Arnold et al. 2015; Junuzović‐Žunić et al. 2023; Lee 2023; Li and Arnold 2015) and could constitute further evidence of regional or cultural differences as one of the most important determinants of attitudes toward stuttering (Abdalla and St. Louis 2012; Irani et al. 2012; St. Louis and Roberts 2013). Another reason for these better attitudes could be due to the inclusion in our study, although in a small proportion, of School Counsellors and School‐Based Speech‐Language Pathologists and Special Education Teachers, in addition to other studies’ traditionally included teaching roles (e.g., Early Childhood Education, Primary and High School Education).

Compared to the *POSHA–S* database, our data showed that teachers in Spain were especially positive on the *Beliefs* subscore and better than the database average on the *Self‐Reactions* subscore. This finding supports studies that reported a positive relationship between the *Beliefs* and *Self‐Reactions* subscores (Arnold and Li 2016, St. Louis 2024b).

Regarding the *Beliefs* subscore, although our teachers’ ratings of the component of *Traits/Personality* and all its items, were more positive than those in the *POSHA–S* database, teachers still substantially shared common prejudices about students and others who stutter as being nervous or shy. These traits are usually considered by teachers as the result of their stuttering, leading teachers not to consider stuttering a problem in the case of other students who participate or ask questions regularly in the classes (Adriaensens and Struyf 2016). Our sample attributed a genetic cause to stuttering, which reflects current research consensus (Adriaensens and Struyf 2016; Hearne et al. 2021; Irani et al. 2012; Lefort et al. 2021; Panico et al. 2018; Yeakle and Cooper 1986), to a lesser extent than other causes. We submit that this may be due to a resistance related to incorrect notions about non‐modifiable genetic factors and lack of knowledge about permeable gene‐environment interactions (Condit 2010). Moreover, this misconception could have harmful effects on students who stutter, since external locus of control, such as genetic and biological causes have been related to more positive attitudes toward stuttering in terms of less likelihood that people who stutter are to be blamed for their stuttering and favouring better *Accommodating/Helping Reactions* (Abasi 2022; Arnold and Li 2016; Boyle 2016). Finally, although teachers in Spain disregarded in a greater proportion incorrect aetiologies of stuttering comparing to the *POSHA–S* database, a considerable proportion of them still thought that stuttering is learned, caused by an emotionally traumatic experience, or is a symptom of a virus or disease.

In the *Helping* component, although teachers in Spain were more likely to believe that professionals such as speech‐language pathologists and other people who stutter can help in the treatment of stuttering compared to the *POSHA–S* database participants, they still considered doctors as adequate to deal with this problem, which could be interpreted as a lack of knowledge. Finally, the scores at the *Potential* component are revealing. Although teachers conceived people who stutter can do any job they want, they limited a stuttering person's potential when it comes to jobs that are demanding. This finding is worrying since this component has been associated with *Accommodating/Helping Reactions* and *Social Distance/Sympathy* (Arnold and Li 2016) and is related to role‐entrapment, according to which, teachers may be acting upon paternalism by their own idea of what is best for people who stutter without consulting them (Irani et al. 2009).

Regarding the *Self‐Reactions subscore*, in the *Accommodating/Helping* component, teachers in Spain held worse attitudes on the item ‘If I was talking with a person who stutters, I would make a joke’, than other studies reporting even less positive OSSs (e.g., Abdalla and St. Louis 2012). Our Spanish sample seemed more prone to use humour, again reflecting a possible lack of knowledge. Furthermore, although the teachers had better attitudes on the remaining items, a substantial proportion would tell a person who stutters to slow down or relax. This also holds true in the *Social Distance/Sympathy* component, in which, although they were more positive compared with the *POSHA–S* database, many teachers felt uncomfortable when talking with someone who stutters. Most strikingly, our sample was among the worst in the *POSHA–S* database regarding the component *Knowledge Source*, in which teachers in Spain seemed to totally lack sources of information about stuttering. Overall, the lack of knowledge and sources of information about stuttering seems incongruent with rising prevalence rates of students who stutter (Craig and Tran 2005). On the contrary, it would be expected that as a problem becomes more important, the attention given to it in terms of the number of sources of information and knowledgeable teachers should be proportionate. This result could be indicative of the little attention paid to this human attribute, as well as being a contributor to teachers’ belief that, in the case of stuttering, it is better not to react, not to pay attention or to talk about it at all (Adriaensens and Struyf 2016). It is also congruent with SWS regard of school as less supportive than family, workers, bosses or the media (St. Louis et al. 2017).

### 4.2 Comparisons of Teachers' Attitudes in Spain Toward Stuttering According to Sociodemographic Variables

Apart from the description of the characteristics of our sample, the literature shows interest in considering the associative value of some variables. Up to now, there has been a discrepancy about the role of sex. Our results align with other studies, that is, partial or no relationship at all (Arnold et al. 2015; Chon 2016; Lefort et al. 2021; Li and Arnold 2015). This apparent lack of agreement toward the role of sex has been justified by the diverse occupations of the samples or differences depending on the sex of the people who stutter (St. Louis 2012).

As to the variables age, years of education and years of teaching experience, in general, the observed correlations with the different subscores and components of the *POSHA–S* were low. That said, significant correlations occurred in some cases. Specifically, the component *Knowledge/Experience* showed a positive relationship with age and teaching experience. Hence, in line with other studies (Almudhi 2022; Arnold et al. 2015; Chon 2016; Li and Arnold 2015), as teachers become older and have more years of teaching experience, they report more knowledge about stuttering and know more people who stutter and have more personal experience with them. However, age did not always exert a positive influence on attitudes toward stuttering, as could be seen from the significant relationship found with the *Helping* component, wherein older teachers held less accurate views about which people should help people who stutter. In the case of the *Causation* component, years of education were also significantly correlated with less accurate stuttering attitudes, that is, ignoring the relevance of genetic contributions, as seen in other studies (Arnold et al. 2015; Almudhi 2022; Lee 2023; Lefort et al. 2021; Li and Arnold 2015; Valente et al. 2017). This could imply a reluctance to consider constitutionally based causal factors favouring a priori (or more favourable) aetiology such as modifiable environmental factors, as mentioned above.

Regarding the variable of teaching experience with students who stutter, a large proportion of the teachers in our sample reported having no experience teaching students who stutter, which is considered a limiting factor in the development of accurate or sensitive attitudes toward students' stuttering (Abdalla and St. Louis 2012; Boyle 2016; Irani et al. 2012; Lee 2023; Yeakle and Cooper 1986). In fact, in our study, it was observed that those teachers who had no teaching experience with students with stuttering had less accurate or sensitive scores on *OSS, Self‐Reactions*, *Knowledge/Experience* and *Source of Knowledge*. The same pattern, including also the *Causation* and *Accommodating/Helping* components, was obtained for those teachers who had not received training in stuttering, who were most of the participants, although they valued the importance of receiving such training. According to the literature, prior knowledge and training on stuttering play an important role in teachers' perceptions of people who stutter (Arnold and Li 2016; Crowe and Walton 1981; Daniels et al. 2011; Hearne et al. 2021; Panico et al. 2018; Woods and Williams 1976; Yeakle and Cooper 1986). On the other hand, as expected, those participants who self‐identified as people with stuttering scored more accurately on OSS and Beliefs, but not on *Self‐Reactions*. This could reflect a pattern by which external beliefs about other people who stutter are more positive than when it comes to internal self‐reactions about own behaviours, feelings, thoughts and knowledge in front of people who stutter (St. Louis et al. 2016). Finally, if we consider the ‘Current teaching role’, specialist teachers, such as School Counsellors, School‐Based Speech‐Language Pathologists (SLPs) and Special Education Teachers, showed better attitudes toward stuttering than preschool, primary or higher education teachers. Although this finding may seem expected, this has not always been the case (Lee 2014; Maviş et al. 2013).

### 4.3 Explanatory Power of the Sociodemographic Variables on OSS, Beliefs and Self‐Reactions

Finally, the variables that showed the strongest correlation with more positive attitudes toward stuttering and were included in the explanatory model, were the ones that are most specifically related to experience and knowledge about stuttering, that is, ‘Received training in stuttering’, ‘Teaching experience with students who stutter’, ‘Self‐identifying as a person who stutters’ and ‘Current teaching role’. Accordingly, training in stuttering and teaching experience with stuttering have been shown to be predictors of both the *OSS* and the *Self‐Reactions* subscore. Another important predictor of *Self Reactions* was the above‐mentioned ‘Current teaching role’. Hence, together with extensive literature as we analyzed before, we can say that receiving training and having experience with stuttering are paramount factors in tackling stuttering by making teachers better able to help people who stutter. Self‐Identifying as a person who stutters was also present in the model of the *OSS* and was the only variable that remained significant in the model for the *Beliefs* subscore. We hypothesized that teachers who self‐identified with people who stutter were better able to resist prejudices about traits and causes of stuttering, although they still found it hard when it comes to transferring these more positive beliefs to their own *Self Reactions* (St. Louis et al. 2016).

### 4.4 Limitations, Strengths and Future Directions

Our study had some limitations as well as strengths. The unequal sample sizes for the various subsamples was a limitation; however, our overall sample size was similar to other related studies (Abdalla and St. Louis 2012; Arnold et al. 2015; Irani et al. 2009, Irani et al. 2012; Li and Arnold 2015; Panico et al. 2018) and larger than the mean in the *POSHA–S* database, that is, 118 (St. Louis 2024a). Another limitation concerns the mode of sampling, that is, a convenience sample rather than a probability sample (e.g., Valente et al. 2017). These and the self‐reported nature of the different measures may have affected inference and generalization. The other limitation was that a bigger proportion of teachers in our sample belonged to the higher education level, although according to other studies, this may not be relevant since they hold similar views Panico; Werle and Byrd 2021). On the other hand, our study had the strength of considering a hitherto unstudied population, namely, teachers in Spain. To our knowledge, this was the first study that aimed to analyze the explanatory power of sociodemographic variables in the attitudes toward stuttering phenomena in this population. Future research should include bigger probability sampling, as well as longitudinal and qualitative measures (Abrahams et al. 2016; Lefort et al. 2021; Li and Arnold 2015). Moreover, from our perspective, additional comparative regional samples of more modest size would be required in countries that have not yet been studied, or in those where the literature suggests potential differences. This line of research could be helpful in clarifying the rationale for the better attitudes observed in our sample of teachers in Spain.

## 5 Conclusion

As a conclusion, although more positive attitudes toward stuttering were found in the case of teachers in Spain, prejudices did exist, and consequently, people who stutter quality of life may be compromised. Misconceptions concerning the ignorance about the genetic causes of stuttering, the regard of people who stutter as nervous and shy, the perceived limited potential of people who stutter to implement demanding jobs, behaviours such as saying ‘slow down’ or ‘relax’ when talking with people who stutter, or the lack of sources of information, have for long been observed in the stuttering literature, as well as its damaging consequences (Plexico et al. 2013). The practical implications that emerge from our results are that training and experience are important predictors of stuttering attitudes. This finding involves the need to escalate stuttering in the agenda of SEN priorities by education authorities and to develop training programs to counteract teachers’ beliefs about stuttering that take into consideration cultural variations (Abdalla and St. Louis 2012; Abrahams et al. 2016; Adriaensens and Struyf 2016) and that should include evidence‐based knowledge about stuttering as well as sharing experience with people who stutter (Arnold and Li 2016; Junuzović‐Žunić et al. 2023).

## Ethics Statement

The study was carried out in accordance with national and international ethical standards (Helsinki and Tokyo Conventions) and was approved by the Aragón Research Ethics Committee (PI23/258).

## Conflicts of Interest

The authors declare that there is no conflict of interest in the terms specified by this journal.

## Supporting information




**Supplementary Table** **1**. Comparisons of Spanish teachers' attitudes towards stuttering according to sex.; **Supplementary Table** **2**. Comparisons of Spanish teachers' attitudes towards stuttering according to age, years of completed studies and years of teaching experience; **Supplementary Table 3**. Comparisons of Spanish teachers' attitudes towards stuttering according to teaching experience with PWS, having received training, identifying themselves as stutterers; **Supplementary Table 4**. Comparisons of Spanish teachers' attitudes towards stuttering according to current teaching

## Data Availability

The data that support the findings of this study are available from the corresponding author upon reasonable request.
