# Supplementary material for: Analyzing the Attitudes of Teachers in Spain Toward Stuttering
Source: Int J Lang Commun Disord. 2026 Mar 26;61(3):e70223. doi: 10.1111/1460-6984.70223 (PMC13022064; doi:10.1111/1460-6984.70223)
Supplement: Supplementary file 1 — Supplementary Table 1 . Comparisons of Spanish teachers' attitudes towards stuttering according to sex.; Supplementary Table 2 . Comparisons of Spanish teachers' attitudes towards stuttering according to age, years of completed studies and years of teaching experience; Supplementary Table 3. Comparisons of Spanish teachers' attitudes towards stuttering according to teaching experience with PWS, having received training, identifying themselves as stutterers; Supplementary Table 4. Comparisons of Spanish teachers' attitudes towards stuttering according to current teaching [file JLCD-61-0-s001.docx]

**Supplementary material 1**

**Supplementary Table 1.** Comparisons of Spanish teachers' attitudes towards stuttering according to sex.

|  | **Sex** | | | | |
| --- | --- | --- | --- | --- | --- |
|  | t/U | df | Mean/mean rank men | Mean/mean rank women | *p* |
| **Overall Stuttering Score (OSS)** | - 0.606 | 248 | 31.42 | 32.61 | 0.545 |
| **Beliefs** | 7970 | 248 | 117.42 | 130.13 | 0.181 |
| **Traits/Personality** | 7920 | 248 | 117.97 | 129.81 | 0.197 |
| **Help From** | 7080 | 248 | 127.20 | 124.53 | 0.762 |
| **Cause** | 8625.50 | 248 | 110.21 | 134.25 | **0.009** |
| **Potential** | 6860 | 248 | 129.62 | 123.14 | 0.463 |
| **Self-Reactions** | -.048 | 248 | 11.27 | 11.41 | 0.962 |
| **Accommodating/Helping** | 8337.50 | 248 | 113.38 | 132.44 | **0.040** |
| **Social Distance/Sympathy** | 6950.5 | 248 | 128.62 | 123.71 | 0.605 |
| **Knowledge/Experience** | 6787 | 248 | 130.42 | 122.69 | 0.415 |
| **Knowledge Source** | 7498.5 | 248 | 122.60 | 127.16 | 0.626 |

**Supplementary Table 2.** Comparisons of Spanish teachers' attitudes towards stuttering according to age, years of completed studies and years of teaching experience.

|  | **Age** | **Years of completed studies** | **Years of teaching experience** |
| --- | --- | --- | --- |
| **Overall Stuttering Score (OSS)** | -0.035 | -0.020 | 0.051 |
| **Beliefs** | -0.072 | 0.010 | -0.026 |
| **Traits/Personality** | -0.012 | 0.000 | -0.004 |
| **Help From** | **-0.185**** | 0.066 | -0.106 |
| **Cause** | 0.041 | **-0.156*** | 0.063 |
| **Potential** | 0.043 | 0.089 | 0.060 |
| **Self-Reactions** | 0.006 | -0.039 | 0.088 |
| **Accommodating/Helping** | -0.074 | -0.039 | -0.004 |
| **Social Distance/Sympathy** | -0.025 | -0.015 | 0.026 |
| **Knowledge/Experience** | **0.156*** | -0.067 | **0.175**** |
| **Knowledge Source** | -0.091 | 0.022 | -0.026 |

**Note:** *p < 0.050; **p < 0.010.

**Supplementary Table 3.** Comparisons of Spanish teachers' attitudes towards stuttering according to teaching experience with PWS, having received training, identifying themselves as stutterers.

| **Teaching experience with PWS** | | | | | **Having received training** | | | | | **Identifying themselves as stutterers** | | | | |  |
| --- | --- | --- | --- | --- | --- | --- | --- | --- | --- | --- | --- | --- | --- | --- | --- |
|  | t/U | df | Mean/mean rank | | *p* | t/U | df | Mean/mean rank | | *p* | t/U | df | Mean/mean rank | | *p* |
| **Overall Stuttering Score (OSS)** | -3.621 | 248 | No | 29.87 | **< 0.001** | -5.075 | 248 | No | 29.78 | **< 0.001** | -2.412 | 248 | No | 31.74 | **0.017** |
|  |  |  | Yes | 36.99 |  |  |  | Yes | 40.86 |  |  |  | Yes | 43.82 |  |
| **Beliefs** | 7281.5 | 248 | No | 122.91 | 0.414 | 6088.5 | 248 | No | 121.44 | 0.090 | 1717.5 | 248 | No | 122.87 | **0.003** |
|  |  |  | Yes | 130.90 |  |  |  | Yes | 140.25 |  |  |  | Yes | 195.83 |  |
| **Traits/Personality** | 7143 | 248 | No | 123.73 | 0.563 | 5449 | 248 | No | 124.70 | 0.730 | 1374.5 | 248 | No | 124.30 | 0.158 |
|  |  |  | Yes | 129.19 |  |  |  | Yes | 128.41 |  |  |  | Yes | 157.72 |  |
| **Help From** | 6989 | 248 | No | 124.64 | 0.771 | 5683 | 248 | No | 123.51 | 0.370 | 1445.5 | 248 | No | 124.00 | 0.067 |
|  |  |  | Yes | 127.28 |  |  |  | Yes | 132.74 |  |  |  | Yes | 165.61 |  |
| **Cause** | 7580.5 | 248 | No | 121.14 | 0.158 | 6470 | 248 | No | 119.49 | **0.010** | 1231.5 | 248 | No | 124.89 | 0.479 |
|  |  |  | Yes | 134.59 |  |  |  | Yes | 147.31 |  |  |  | Yes | 141.83 |  |
| **Potential** | 6339.5 | 248 | No | 128.49 | 0.309 | 5517 | 248 | No | 124.35 | 0.606 | 1427 | 248 | No | 124.08 | 0.083 |
|  |  |  | Yes | 119.27 |  |  |  | Yes | 129.67 |  |  |  | Yes | 163.56 |  |
| **Self-Reactions** | -4.430 | 248 | No | 7.18 | **< 0.001** | -5.304 | 248 | No | 7.62 | **< 0.001** | -1.224 | 248 | No | 11.02 | 0.222 |
|  |  |  | Yes | 20.07 |  |  |  | Yes | 24.90 |  |  |  | Yes | 20.29 |  |
| **Accommodating/Helping** | 7652.5 | 248 | No | 120.72 | 0.123 | 7077.5 | 248 | No | 116.39 | **< 0.001** | 1229 | 248 | No | 124.90 | 0.488 |
|  |  |  | Yes | 135.48 |  |  |  | Yes | 158.56 |  |  |  | Yes | 141.56 |  |
| **Social Distance/Sympathy** | 7364.5 | 248 | No | 122.42 | 0.330 | 5542.5 | 248 | No | 124.22 | 0.594 | 1058.5 | 248 | No | 125.61 | 0.903 |
|  |  |  | Yes | 131.92 |  |  |  | Yes | 130.14 |  |  |  | Yes | 122.61 |  |
| **Knowledge/Experience** | 9419 | 248 | No | 110.27 | **< 0.001** | 6575 | 248 | No | 118.95 | **0.006** | 1159 | 248 | No | 125.19 | 0.726 |
|  |  |  | Yes | 157.28 |  |  |  | Yes | 149.26 |  |  |  | Yes | 133.78 |  |
| **Knowledge Source** | 8001.5 | 248 | No | 118.65 | **0.028** | 7578.5 | 248 | No | 113.83 | **< 0.001** | 1382 | 248 | No | 124.27 | 0.156 |
|  |  |  | Yes | 139.78 |  |  |  | Yes | 167.84 |  |  |  | Yes | 158.56 |  |

**Supplementary Table 4.** Comparisons of Spanish teachers' attitudes towards stuttering according to current teaching role.

|  | **Current teaching role** | | | | | |
| --- | --- | --- | --- | --- | --- | --- |
|  | F/H | df | Mean/mean rank | | *p* | Post hoc results |
| **Overall Stuttering Score (OSS)** | 7.818 | 2 | A. Other type | 30.82 | **< 0.001** | **A** ≠ **B (p = 0.038)** |
|  |  |  | B. Guidance counselor | 40.86 |  | **A** ≠ **C (p = 0.004)** |
|  |  |  | C. Specialist | 42.54 |  | B = C (p = 1.000) |
| **Beliefs** | 1440 | 2 | A. Other type | 123.44 | 0.487 | A = B (p = 1.000) |
|  |  |  | B. Guidance counselor | 138.82 |  | A = C (p = 1.000) |
|  |  |  | C. Specialist | 141.03 |  | B = C (p = 1.000) |
| **Traits/Personality** | .664 | 2 | A. Other type | 124.90 | 0.717 | A = B (p = 1.000) |
|  |  |  | B. Guidance counselor | 139.82 |  | A = C (p = 1.000) |
|  |  |  | C. Specialist | 121.38 |  | B = C (p = 1.000) |
| **Help From** | 1301 | 2 | A. Other type | 124.86 | 0.522 | A = B (p = 1.000) |
|  |  |  | B. Guidance counselor | 115.86 |  | A = C (p = 0.857) |
|  |  |  | C. Specialist | 141.68 |  | B = C (p = 0.956) |
| **Cause** | 7.689 | 2 | A. Other type | 121.66 | **0.021** | A = B (p = 1.000) |
|  |  |  | B. Guidance counselor | 130.89 |  | **A** ≠ **C (p = 0.017)** |
|  |  |  | C. Specialist | 170.56 |  | B = C (p = 0.356) |
| **Potential** | 0.065 | 2 | A. Other type | 125.10 | 0.968 | A = B (p = 1.000) |
|  |  |  | B. Guidance counselor | 127.93 |  | A = C (p = 1.000) |
|  |  |  | C. Specialist | 128.18 |  | B = C (p = 1.000) |
| **Self-Reactions** | 9.623 | 2 | A. Other type | 9.11 | **< 0.001** | **A ≠ B (p = 0.012)** |
|  |  |  | B. Guidance counselor | 26.37 |  | **A ≠ C (p = 0.002)** |
|  |  |  | C. Specialist | 27.98 |  | B = C (p = 1.000) |
| **Accommodating/Helping** | 10.445 | 2 | A. Other type | 120.30 | **0.005** | A = B (p = 0.433) |
|  |  |  | B. Guidance counselor | 148.79 |  | **A ≠ C (p = 0.009)** |
|  |  |  | C. Specialist | 173.26 |  | B = C (p = 1.000) |
| **Social Distance/Sympathy** | 0.555 | 2 | A. Other type | 125.91 | 0.757 | A = B (p = 1.000) |
|  |  |  | B. Guidance counselor | 112.50 |  | A = C (p = 1.000) |
|  |  |  | C. Specialist | 130.88 |  | B = C (p = 1.000) |
| **Knowledge/Experience** | 5.432 | 2 | A. Other type | 121.83 | 0.066 | A = B (p = 0.909) |
|  |  |  | B. Guidance counselor | 164.64 |  | A = C (p = 0.094) |
|  |  |  | C. Specialist | 140.53 |  | B = C (p = 1.000) |
| **Knowledge Source** | 21.190 | 2 | A. Other type | 117.71 | **< 0.001** | **A ≠ B (p = 0.001)** |
|  |  |  | B. Guidance counselor | 180.64 |  | **A ≠ C (p = 0.004)** |
|  |  |  | C. Specialist | 180.44 |  | B = C (p = 1.000) |
